# Efficacy of polymyxin B-immobilized fiber hemoperfusion for patients with septic shock caused by Gram-negative bacillus infection

**DOI:** 10.1371/journal.pone.0173633

**Published:** 2017-03-30

**Authors:** Nobuyuki Saito, Kazuhiro Sugiyama, Testu Ohnuma, Takashi Kanemura, Michitaka Nasu, Yuya Yoshidomi, Yuta Tsujimoto, Hiroshi Adachi, Hiroyuki Koami, Aito Tochiki, Kota Hori, Yukiko Wagatsuma, Hisashi Matsumoto

**Affiliations:** 1 Shock and Trauma Center, Nippon Medical School Chiba Hokusoh Hospital, Chiba, Japan; 2 Faculty of Medicine, Department of Clinical Trial and Clinical Epidemiology, University of Tsukuba, Ibaraki, Japan; 3 Department of Emergency and Critical Care Medicine, Tokyo Metropolitan Bokutoh Hospital, Tokyo, Japan; 4 Intensive Care Unit, Department of Anesthesiology, Saitama Medical Center, Jichi Medical University, Saitama, Japan; 5 Department of Emergency and Critical Care Medicine, National Disaster Medical Center, Tokyo, Japan; 6 Department of Emergency and Critical Care Medicine, Urasoe General Hospital, Okinawa, Japan; 7 Department of Emergency Medicine, Saga-Ken Medical Center Koseikan, Saga, Japan; 8 Department of Emergency Medicine, Yamagata Prefectural Central Hospital, Yamagata, Japan; 9 Department Intensive Care Medicine, Iizuka Hospital, Fukuoka, Japan; 10 Advanced Emergency Care Center, Saga University Hospital, Saga, Japan; 11 Department of Emergency and Critical Care Medicine, Tsukuba Medical Center Hospital, Ibaraki, Japan; 12 Department of Emergency and Critical Care Medicine, Japanese Red Cross Kumamoto Hospital, Kumamoto, Japan; Bambino Gesù Children's Hospital, ITALY

## Abstract

Septic shock-associated mortality in intensive care units (ICUs) remains high, with reported rates ranging 30–50%. In particular, Gram-negative bacilli (GNB), which induce significant inflammation and consequent multiple organ failure, are the etiological bacterial agent in 40% of severe sepsis cases. Hemoperfusion using polymyxin B-immobilized fiber (PMX), which adsorbs endotoxin, is expected to reduce the inflammatory sepsis cascade due to GNB. However, the clinical efficacy of this treatment has not yet been demonstrated. Here, we aimed to verify the efficacy of endotoxin adsorption therapy using PMX through a retrospective analysis of 413 patients who received broad spectrum antimicrobial treatment for GNB-related septic shock between January 2009 and December 2012 in 11 ICUs of Japanese tertiary hospitals. After aligning the patients' treatment time phases, we classified patients in two groups depending on whether PMX hemoperfusion (PMXHP) therapy was administered or not within 24 hours after ICU admission (PMXHP group: n = 134, conventional group: n = 279). The primary study endpoint was the mortality rate at 28 days after ICU admission. The mean age was 72.4 (standard deviation: 12.6) years, and the mean Sequential Organ Failure Assessment score at ICU admission was 9.9 (3.4). The infection sites included intra-abdominal (38.0%), pulmonary (18.9%), and urinary tract (32.2%), and two thirds of all patients had GNB-related bacteremia. Notably, the mortality at 28 days after ICU admission did not differ between the groups (PMXHP: 29.1% vs. conventional: 29.0%, P = 0.98), and PMXHP therapy was not found to improve this outcome in a Cox regression analysis (hazard ratio = 1.16; 95% confidence interval, 0.81–1.64, P = 0.407). We conclude that PMX-based endotoxin adsorption within 24 hours from ICU admission was not associated with mortality among patients with septic shock due to GNB. Trial registration: University Hospital Medical Information Network Clinical Trial Registry (UMIN-CTR ID: UMIN000012748).

## Introduction

The incidence of septic shock (SS), a critical and potentially fatal illness characterized by an excessive biological reaction against an infections pathogenic microorganism, is increasing worldwide [1]. Since 2004, international guidelines for the management of severe sepsis and septic shock (the Surviving Sepsis Campaign) have advanced the standardization of primary care for sepsis [2], and SS-related mortality rates have decreased steadily in intensive care units (ICUs) [3]. However, the clinical outcomes of SS vary widely, with reported mortality rates ranging from 20% to 50% [3,4].

Currently, Gram-negative bacilli (GNB) are the etiological bacterial agent in 40% of SS cases [1]; these pathogens are known to cause excessive inflammatory reactions that can lead to multiple organ failure [5,6]. Unfortunately, GNB is also a major causative organism of nosocomial infections, and the resulting increase in drug resistance has led to treatment challenges [7]. The GNB outer membrane component endotoxin is a well-known, typical pathogen-associated molecular component that can induce inflammation [8], and as early as a few decades ago, GNB-induced SS was described as endotoxin shock and considered to be a more critical condition [9,10]. These findings have led to considerable research regarding the potential of endotoxin as a therapeutic target [11].

In the 1980s, this endotoxin-related research led to the development of a polymyxin B-immobilized fiber column (PMX: Toraymyxin®; Toray Medical Co., Ltd., Tokyo, Japan), which utilizes the ability of polymyxin B to bind lipid A within the major endotoxin effector site. The endotoxin adsorption efficacy of PMX, which has been used in clinical applications in Japan since 1994, has been demonstrated both in vitro and in vivo, and this material has since been used with the hope that it could suppress the GNB-related inflammatory cascade [12]. More recently, PMX has been used to treat intra-abdominal infection in several countries.

Although high serum endotoxin levels are associated with organ dysfunction in patients with SS, there is no clear evidence regarding the clinical effect of endotoxin adsorption therapy with PMX hemoperfusion (PMXHP) on survival [11,13,14]. In a 2007 systematic review of PMXHP [15], Cruz et al. reported that the arterial pressure and pulmonary oxygenation (PaO2/FiO2 ratio) improved and mortality decreased with the implementation of PMXHP (odds ratio [OR] = 0.53, 95% confidence interval: 0.43–0.65). In contrast, recent publications by Cruz et al. in 2009 [16] and Payen et al. in 2015 [17] reported no significant decreases in mortality among patients with abdominal sepsis. Similar results were observed among Japanese patients with lower gastrointestinal perforation in a propensity-matched analysis of nationwide inpatient insurance data [18]. Recently, the EUPHRATES trail (CrinicalTrial.gov. identifier NCT01046669) as phase III trial for PMXHP therapy has been initiated in North America. However, all of these previous reports were limited by a lack of certainty regarding the GNB infection statuses of all target patients.

We conducted a multi-center study with the intent to verify the following hypothesis: after achieving infection site control and implementing broad-spectrum antibacterial treatment, the 28-day mortality rate would improve with the addition of PMXHP within 24 hours from ICU admission as an adjuvant therapy in patients with SS due to GNB.

## Methods

### Study setting

We conducted a retrospective study at 11 ICUs of Japanese tertiary hospitals. Before data collection, the study protocol was reviewed and approved by the ethics committee of Nippon Medical School Hokusoh Hospital. The trial was registered in the University Hospital Medical Information Network Clinical Trial Registry (UMIN-CTR ID: UMIN000012748).

### Data collection

For this study, we enlisted 11 tertiary care facilities with the ability to provide sufficient treatment to critically ill patients, as well as resident education programs. All of those facilities deployed electronic medical record systems for the collection of information. Each investigator was provided a comprehensive manual that described the data collection requirements and definitions of variables. Case report forms were uploaded to the study website via the internet. Case registration was mandatory to ensure correct alignment of the treatment order and time-phase and adjustment of confounding factors. Data management was performed at an independent data center at the University of Tsukuba (CREIL Center, Ibaraki, Japan).

The inclusion criteria were admission to an ICU between January 2009 and December 2012, age ≥18 years, and SS resulting from GNB, as detected from cultured clinical specimens. Sepsis was diagnosed according to the 2008 Surviving Sepsis Campaign Guidelines [19]. In addition, SS was defined as hypotension (systolic blood pressure <90 mmHg) at the start of the medical intervention or an elevated lactate level (>4 mmol/L). Microbial confirmation of GNB required the isolation of pathogens from any cultured clinical specimens. This study excluded patients with a non-resuscitation status.

As a premise, patients received broad-spectrum antimicrobial treatment and, if needed, surgical intervention for source control before ICU admission. After aligning each patient's treatment time phase, we classified patients into two groups depending on the administration of PMXHP treatment within 24 hours from ICU admission (PMXHP group: n = 134, conventional group: n = 279).

To compare patients’ conditions before PMXHP therapy, the following information was collected: age, sex, severity of illness (e.g., Acute Physiology and Chronic Health Evaluation II [APACHE] II score [20], Sequential Organ Failure Assessment [SOFA] score [21]) pre-existing disease, comorbidities upon ICU admission (e.g., all grades of acute respiratory distress syndrome [ARDS] defined according to the criteria of the Berlin definition [22], all grades of acute kidney injury [AKI] defined according to the RIFLE criteria [23], disseminated intravascular coagulopathy [DIC] diagnosed using the Japanese Association for Acute Medicine DIC scores [24], acute coronary syndrome/stroke diagnosed by a vascular lesion specialist, intraperitoneal abscess diagnosed from imaging findings), type of infection (e.g., community acquired, hospital acquired, healthcare acquired), site of infection (e.g., pulmonary, intra-abdominal, urinary, soft tissue), vital signs, and laboratory data at the beginning of the medical intervention. Details of defined cultures were collected separately from blood and local samples. Information about treatment for comorbidities after ICU admission and specific drug usage was also collected.

### Implementation of PMXHP therapy

Decisions regarding PMXHP therapy were left to each facility. The most common indication for PMX treatment was SS with suspicion of GNB infection regardless of the site. Japanese public insurance allows the performance of 2-hour direct hemoperfusion sessions with heparin administration as a basic PMXHP protocol. In the current study, this protocol has been adjusted for anti-coagulant drugs and the duration of direct hemoperfusion at each facility. In addition, further options could be added to a subsequent session of PMXHP after completing the initial session. Data regarding the implementation of PMXHP, vital signs, and laboratory data of the patient before and after the implementation PMXHP therapy were collected.

### Outcomes

The primary endpoint was the mortality rate at 28 days after ICU admission, and the secondary endpoints were the mortality rate at hospital discharge, duration of mechanical ventilation, length of ICU stay, and length of hospital stay. In addition, outcome-free days (e.g., ventilator-free days, ICU-free days) were determined to minimize survivor bias. Comorbidities after ICU admission, including ARDS, AKI, DIC, acute coronary syndrome, stroke, and intraperitoneal abscess, were also recorded as clinical outcomes.

### Sample size and statistical methods

Based on previous studies [3–5,16], we assumed that in order to be clinically meaningful, the assumed mortality rate of 40% at 28 days for the target patient population would need to be reduced to 25% after implementing PMXHP therapy. Assuming that PMX intervention was performed in one-third of the target patients, the size needed to test an absolute reduction in mortality at 28 days of 15% (relative reduction of 37%) would be 390 patients (130 for the PMXHP group and 260 for the conventional group) to obtain a nominal two-sided p value of 0.05 and power of 85%.

The groups were compared with respect to demographic and clinical parameters. Significant differences in means, medians, and prevalence estimates were determined using the chi-square test for categorical variables, the t test or Mann–Whitney U test for independent continuous variables, and the Wilcoxon signed rank test for paired continuous variables. P values <0.05 were considered significant. A Cox proportional hazards model adjusted for age, sex, and pre-treatment status was used for the multivariate analysis. All statistical analyses were performed using SPSS, version 23.0 (IBM, Armonk, NY, USA).

## Results

### Patient characteristics

The mean age of patients in this study was 72.4 years (standard deviation: 12.6), and the mean SOFA score upon ICU admission was 9.9 (3.4). Mechanical ventilation was performed in 72.9% of all patients, and a third of patients underwent surgery for source control. The infection sites were intra-abdominal, pulmonary, and urinary tract in 38.0%, 18.9%, and 32.2% of patients, respectively, and two-thirds (67%) of all patients had bacteremia due to GNB.

Table 1 compares patient characteristics, clinical data, and treatments of the PMXHP and conventional groups. Although the PMXHP group was younger than the conventional group, this difference was not significant (P = 0.06). However, the frequencies of comorbid AKI and DIC at the time of ICU admission differed significantly between the two groups (P = 0.01 and <0.01, respectively), and the frequency of mechanical ventilation was significantly higher in the PMXHP group (87.3% vs. 65.9% for the conventional group).

**Table 1:** Comparison of Patient Characteristics between PMXHP and Conventional Groups

	PMXHP group	Conventional group	P value
	n = 134	n = 279	
Age, years	70 ± 13	73 ± 12	0.06
Male / female	63 / 71	153 / 126	0.13
Pre-existing disease			
Chronic heart failure	11 (8.2%)	27 (9.7%)	0.62
Ischemic heart disease	15 (11.2%)	25 (9.0%)	0.47
Chronic obstructive pulmonary disease	3 (2.2%)	13 (4.7%)	0.23
Liver cirrhosis	9 (6.7%)	13 (4.7%)	0.38
Chronic renal failure	3 (2.2%)	10 (3.6%)	0.46
Diabetes	29 (21.6%)	59 (21.1%)	0.90
Cancer	10 (7.5%)	35 (12.5%)	0.12
Comorbidities on ICU admission			
ARDS	21 (15.7%)	42 (15.1%)	0.87
AKI	100 (74.6%)	173 (62.0%)	0.01
DIC	80 (59.7%)	115 (41.2%)	<0.01
Type of infection			
Community acquired	100 (74.6%)	197 (70.6%)	0.39
Hospital acquired	30 (22.4%)	63 (22.6%)	0.96
Healthcare acquired	4 (3.0%)	19 (6.8%)	0.11
Site of infection			
Pulmonary	13 (9.7%)	65 (23.3%)	0.01
Intra-abdominal	68 (50.7%)	89 (31.9%)	<0.01
Urinary	37 (27.6%)	96 (34.4%)	0.16
Soft tissue/skin	6 (4.5%)	12 (4.3%)	0.93
Other/unknown	10 (7.5%)	14 (5.0%)	0.32
Positive blood culture	88 (65.7%)	189 (67.7%)	0.67
Causative pathogens			
*E*. *coli*	69 (51.5%)	135 (48.4%)	0.55
*Pseudomonas aeruginosa*	11 (8.2%)	33 (11.8%)	0.26
*Enterobacter* spp.	14 (10.4%)	26 (9.3%)	0.71
*Klebsiella* spp.	37 (27.6%)	64 (22.9%)	0.30
*Serratia* spp.	5 (3.7%)	7 (2.5%)	0.48
*Acinetobacter* spp.	3 (2.2%)	5 (1.8%)	0.75
*Citrobacter* spp.	5 (3.7%)	8 (2.9%)	0.63
*Haemophilus Influenzae*	1 (0.7%)	10 (3.6%)	0.09
*Other gram-negative rods*	20 (14.9%)	52 (18.6%)	0.35
*Enterococcus* spp.	13 (9.7%)	16 (5.7%)	0.14
*Other gram-positive cocci*	2 (1.5%)	4 (1.4%)	0.96
Multiple pathogens	34 (25.4%)	66 (23.7%)	0.70
Surgery for infection control, total	69 (51.5%)	65 (23.3%)	<0.01
Laparotomy	53 (39.6%)	44 (15.8%)	<0.01
Urological/gynecological surgery	11 (8.2%)	12 (4.3%)	0.10
Extremity/soft tissue surgery	8 (2.9%)	4 (3.0%)	0.94
Vital signs at beginning of treatment			
Mean arterial pressure, mmHg	60 ± 21	59 ± 23	0.98
Heart rate, beats/minute	114 ± 24	111 ± 27	0.35
Respiratory rate, breaths/minute	26 ± 7	26 ± 8	0.71
Glasgow coma scale, points	11 ± 4	11 ± 3	0.56
Laboratory data			
C reactive protein, mg/mL	16.5 ± 11.7	15.9 ± 11.8	0.64
Lactate, mmol/L	5.0 ± 3.8	4.7 ± 3.9	0.53
APACHE II score	26 ± 9	25 ± 9	0.31
SOFA score, total score	10.5 ± 3.8	9.5 ± 3.2	<0.01
Treatment after ICU admission			
Mechanical ventilation	117 (87.3%)	184 (65.9%)	<0.01
Continuous hemodiafiltration	100 (74.6%)	63 (22.6%)	<0.01
Intermittent hemodialysis	2 (1.5%)	6 (2.2%)	0.65
ECMO/PCPS	1 (0.7%)	9 (3.2%)	0.12

Data are presented as median values with interquartile range or as number (%). Pathogens were detected from blood cultures and contained duplications.

ARDS: acute respiratory distress syndrome; AKI: acute kidney injury; DIC: disseminated intravascular coagulation; APACHE; acute physiology and chronic health evaluation; SOFA: sequential organ failure assessment; ECMO: extracorporeal membrane oxygenation; PCPS: percutaneous cardiopulmonary support.

Although the distribution of infection type was homogenous, the distribution of infection site was somewhat heterogeneous. For example, a large proportion of patients in the PMXHP group had intra-abdominal infection, whereas the proportion with pulmonary infection was relatively small. However, both groups had similar frequencies of bacteremia. The causative pathogens were also similar in both groups. Furthermore, surgery for source control was more frequently implemented in the PMXHP group relative to the conventional group. In particular, the proportion of laparotomy in the PMXHP group was significantly higher than that in the conventional group.

Although vital signs and serum lactate level prior to treatment were similar in both groups, the severity of illness, indicated by the SOFA score, was significantly higher in the PMXHP group than that in the conventional group. The continuous hemodiafiltration as renal replacement therapy after ICU admission also differed significantly between the two groups.

### Outcomes

**Fig 1:**
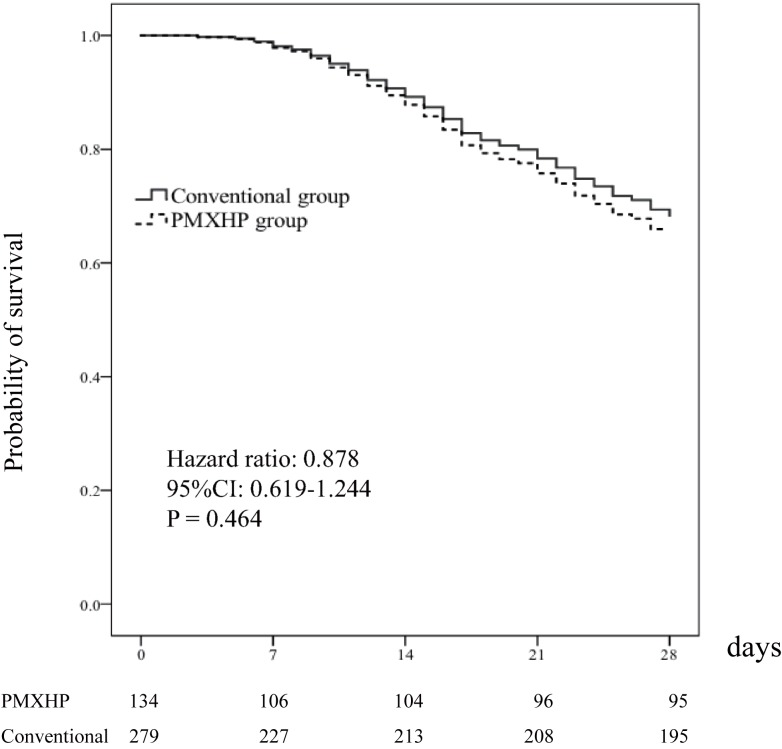
Multivariate Cox Regression Analysis. Patients in the polymyxin B hemoperfusion (PMXHP) group received at least one session of direct PMXHP as adjuvant therapy for septic shock.

**Fig 2:**
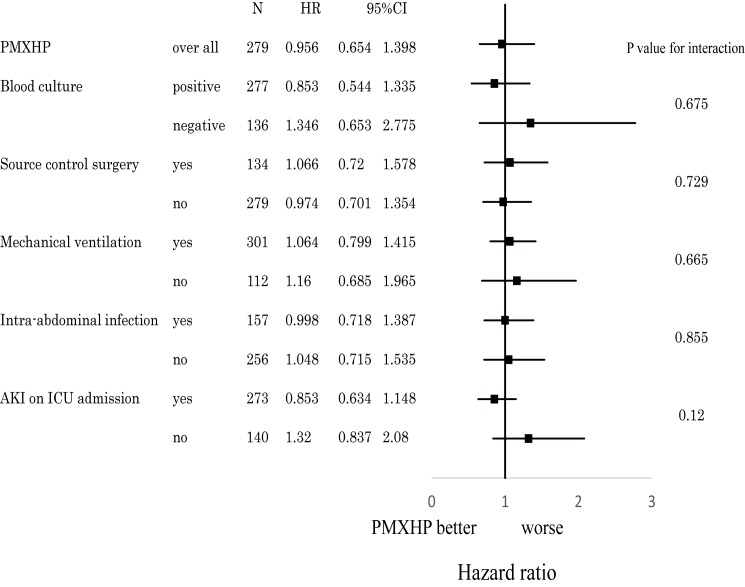
Post-hoc Analysis. The mortality rates at 28 days after ICU admission across the indicated subgroups were defined according to several baseline characteristics. PMXHP: polymyxin B immobilized fiber hemoperfusion, HR: hazard ratio, CI: confidence interval, AKI: acute kidney injury.

The primary endpoint, mortality rate at 28 days after ICU admission, did not differ between the two groups (PMXHP: 29.1% vs. conventional: 29.0%, P = 0.98). For secondary outcomes, duration of mechanical ventilation and ventilator free days were better in the conventional group than the corresponding values in the PMXHP group. The groups differed with regard to additional comorbidities after ICU admission; specifically, ARDS and DIC were more common in the PMXHP group, whereas vascular disease was more common in the conventional group [Table 2]. Fig 1 demonstrates that PMX treatment (hazard ratio = 1.16; 95% confidence interval, 0.81–1.64, P = 0.407) did not improve the study outcome measures, according to a multivariate Cox regression model analysis; in addition, no inter-group differences were observed at hospital discharge. Fig 2 additionally shows a post-hoc subgroup analysis in which we again did not observe differences in efficacy and interactions in any PMXHP subgroup.

**Table 2:** Primary and Secondary Outcomes

	PMXHP group	Conventional group	P value
	n = 134	n = 279	
Primary outcome			
Mortality rate: 28 days after ICU admission	39 (29.1%)	81 (29.0%)	0.98
Secondary outcomes			
Mortality rate: Hospital discharge	51 (38.1%)	69 (34.4%)	0.68
Length of ICU stay, days	8 (4–16)	7 (3–13)	0.11
ICU free days at 28 days	12 (0–20)	15 (0–22)	0.16
Length of hospital stay, days	26 (11–56)	25 (10–43)	0.35
Duration of mechanical ventilation, days	6 (2–13)	3 (0–9)	<0.01
Ventilator free days at 28 days	16 (0–19)	19 (0–24)	0.03
Additional comorbidity after ICU admission			
ARDS	27 (20.1%)	27 (9.7%)	<0.01
AKI	8 (6.0%)	28 (10.0%)	0.17
DIC	31 (23.1%)	37 (13.3%)	0.01
Acute coronary syndrome	1 (0.7%)	9 (3.2%)	0.12
Stroke	1 (0.7%)	12 (4.3%)	0.05
Intraperitoneal abscess	7 (5.2%)	11 (3.9%)	0.55

Data are presented as median values with interquartile range or as number (%).

ICU: intensive care unit; ARDS: acute respiratory distress syndrome; AKI: acute kidney injury; DIC: disseminated intravascular coagulation.

### Hemoperfusion practice

A total of 184 PMXHP sessions were conducted, and the actual median adsorbed time was 144 minutes. The standard 2 hours were not completed in 11 sessions (5.9%), and 10 sessions were canceled due to solidification in the column; in addition, one patient fell into cardiopulmonary arrest and died during PMX treatment. The overall mean arterial pressure increased after PMXHP relative to the pre-treatment value (before PMXHP: 68 [57–80] vs. after PMXHP: 76 [65–87] mmHg, P < 0.01); however, in the initial session, deterioration in blood pressure with or without administration of additional catecholamine was observed in 42.5% (57/134) of patients. No improvements were observed in the PaO2/FiO2 ratio, lactate level, or base deficit (an indicator of circulatory failure; data not shown).

Patients with a rate of change in mean arterial pressure of 10% or more before and after the initial PMXHP session and who were administered vasopressors (n = 69) had a significantly lower mortality rate at ICU discharge than those whose rate of change was less than 10% (n = 65) (unadjusted OR: 0.29 [95% CI: 0.13–0.66], adjusted OR: 0.29 [0.12–0.71]).

## Discussion

In this study, which was conducted in tertiary care hospitals practicing current common procedures for sepsis in Japan, we did not observe an additional clinical benefit of adjuvant PMXHP therapy on mortality after the administration of broad-spectrum antimicrobial treatment and source control among patients with SS, of whom more than 67% presented with GNB bacteremia. Moreover, no subgroup-related differences in efficacy were observed in a post-hoc analysis.

In Japan, PMXHP therapy has been used generally for approximately 20 years [12,15], and protocols have been developed at a number of large-scale facilities. However, the use of PMXHP therapy is left to the physician's preference and facility policy because of its nature as a special and invasive adjunctive therapy. The present study demonstrates the lack of clinical efficacy of PMXHP when administered for typical SS caused by GNB. We suggest that the previously observed effect might be attributable to the Abilene paradox.

In 2015, Payen et al. [17] described a non-significant increase in mortality and no improvement in organ failure following PMXHP vs. conventional treatment for peritonitis-induced SS in the ABDOMIX trial, the latest multicenter randomized control trial in France. Although the results of our study were similar to those of the ABDOMIX trial, the trials differed in terms of focus, as the latter trial addressed SS due to peritonitis in contrast to our study. We note that although most previous reports targeted intra-abdominal infections, GNB infections occur at a much broader range of sites [15,16,25]. In principle, PMXHP is only valid for the treatment of bacteremia caused by GNB. Accordingly, we selected patients with SS caused by GNB with the prior expectation of a high probability of efficacy.

In the ABDOMIX trial [17], randomization was performed at 2 hours after surgery to recruit patients with prolonged SS due to peritonitis. Although we initially confirmed SS, this classification included patients who had emerged from a state of shock at the time of ICU admission, according to resuscitation data from this study. However, 66.4% of patients in the PMXHP group remained in a prolonged state of shock, defined as a mean arterial pressure of ≤65 mmHg or hyperlactatemia upon ICU admission. As a result, the mortality rate at 28 days after ICU admission in our intervention group was 29.1%, similar to that of the ABDOMIX study (27.7%).

We note that serum endotoxin values were not included among the considerations of this study, largely because few facilities have the ability to routinely measure serum endotoxin levels and because no standard method has been set for such measurements. We also did not adopt the endotoxin activity assay for diagnosis of endotoxemia [9,26] as an inclusion criterion because it is not measured in normal practice in Japan. Only patients with bacteriologically proven GNB infection were included, but even GNB infections do not necessarily indicate endotoxemia. In particular, the endotoxin-releasing action varies depending on the type of antibiotics used beforehand. Some classes of β-lactam antibiotics lead to a markedly increased level of free endotoxins, while treatment with aminoglycosides and carbapenems, especially imipenem, produces relatively low amounts of endotoxins [27]. Since we did not specify the type of broad-spectrum antibiotics in this study, we might have included patients with low amounts of free endotoxin despite GNB infection. As a result, the effect of PMXHP treatment might have been diminished. In a clinical trial of anti-endotoxin therapy, the biggest problem is the difficulty of estimating in advance patients for whom treatment would be effective. Indirect diagnosis of endotoxemia by the endotoxin activity assay method looks promising, but it has not yet spread to the general setting. Eventually, the hemodynamic response to PMXHP therapy would have been the only potential prognostic factor in this study.

PMXHP therapy has not been subjected to a randomized trial in Japan or other developed countries. A fair evaluation has thus far been impossible because previous reports from Japan have tended to include considerable bias [15], and only the clinical outcomes of intra-abdominal infection from a nationwide insurance database were reported. In 2014, Iwagami et al. [18] reported the mortality rate at 28 days in the presence or absence of PMXHP using a propensity score matching analysis. Although that report showed a national trend, biological patient information was lacking, and it was necessary to add a supplemental study. In contrast, our study presented clear information about the patients' medical treatment courses, including vital signs. Although the distributions of patient characteristics exhibited considerably heterogeneity, similar to the report by Iwagami et al. [18], the time axis of treatment for SS was maintained in a linear manner. In particular, although the initial complications, infection site, and implementation of surgery varied, we did not observe a causal relationship between the outcomes after adjusting for heterogeneity. Furthermore, we were not able to identify any clinical efficacy of PMXHP, despite performing various stratified post-hoc analyses.

A previous review [15] described improvements in oxygenation and blood pressure as short-term effects of PMXHP. In this study, although the mean arterial pressure increased after the initial PMXHP session relative to the pre-treatment value, the distribution of this effect was quite heterogeneous. However, the prognostic outcome in patients with elevated mean arterial pressure without administration of additional vasopressors was significantly better. In contrast, cases with poor hemodynamic reactivity might exhibit a hindrance to the circulation, thus canceling the effect of PMXHP. On the other hand, we did not obtain positive results regarding the PaO2/FiO2 ratio and blood gas parameters, which raises concerns about the safety of PMXHP therapy.

This study has some limitations of note. First, the study design was observational, and a case-control design was adopted. Because PMX treatment is already commonly used for SS in Japanese healthcare settings, a randomized trial would present ethical challenges. To improve the quality of clinical research, however, the present study involved data collection and management by an independent clinical research organization to verify the likelihood of our interventions and outcomes. Although this was an observational study, the registration data at each facility were regularly monitored, and incompatibilities were coordinated via feedback as well as in a prospective manner. Second, the PMXHP execution rate varied among the participating institutions and its average rate was 32.4% (standard error: 9.7%; range: 0–100%). Although PMXHP itself was feasible at the participating hospitals, the policies regarding PMXHP therapy for SS ranged from completely negative to active affirmation. Despite reflecting actual clinical practice in Japan, there was obvious patient selection bias. In previous epidemiological reports on sepsis [3,4], both mortality rates of intra-abdominal infection and pulmonary infection were high, but urinary tract infections (UTIs) were relatively lower. Although the SS was caused by GNB, the course of treatment varied considerably. The actual mortality rate at 28 days after ICU admission was 35.9% (28/78) for pulmonary infection and 31.8% (50/157) for intra-abdominal infection. On the other hand, mortality rate for UTI was 20.3% (27/133). Although the proportion of UTIs in the PMXHP group was smaller than that in the conventional group, there was no statistical difference, potentially offsetting the efficacy of PMXHP therapy. There was no report on the difference in infection site regarding the effect of PMXHP therapy, and it would not interfere with direct endotoxin removal in circulation in any part of the intruding gate. However, the difference in infection site was related to the frequency of complications [3,4]. Because the combination of ARDS and AKI was a typical prognostic factor, the infection site might have influenced the primary outcome indirectly. Second, the mortality rate used to calculate the sample size differed from the actual number. Consequently, the number of samples in the PMXHP group was smaller than expected, leading to a reduction in the preferred power. Since the elucidation of the pathophysiology of sepsis, the mortality rate associated with sepsis has decreased [4], and a magic bullet in the form of a large difference in mortality might be already out of reach. Accordingly, the hurdles that must be overcome to prove the efficacy of a single adjuvant therapy have increased in size.

In conclusion, no difference in mortality was observed among patients with SS caused by GNB, regardless of the implementation of endotoxin adsorption therapy within 24 hours from ICU admission. Accordingly, reconfirmation of the efficacy and safety of PMX through a multicenter prospective study is essential.

## Supporting information

S1 Dataset This file contains final dataset for SPSS and available with author’s permission. (SAV)
